# Supplementary figures and images for: Genome-Scale Metabolic Models and Machine Learning Reveal Genetic Determinants of Antibiotic Resistance in Escherichia coli and Unravel the Underlying Metabolic Adaptation Mechanisms
Source: mSystems. 2021 Aug 3;6(4):e00913-20. doi: 10.1128/mSystems.00913-20 (PMC8409726; doi:10.1128/mSystems.00913-20)

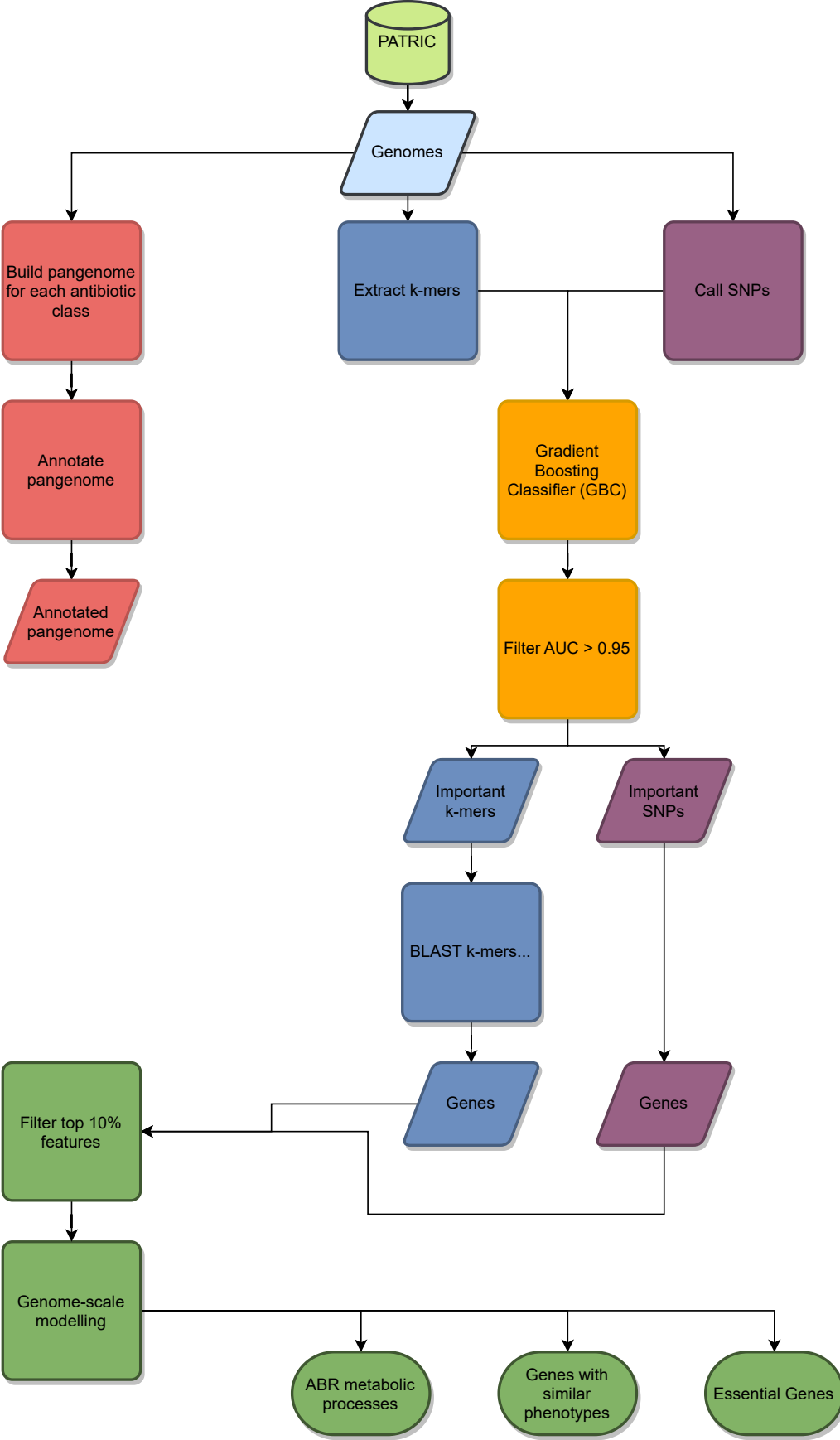

Supplement: FIG S1 [file msystems.00913-20-sf001.pdf]

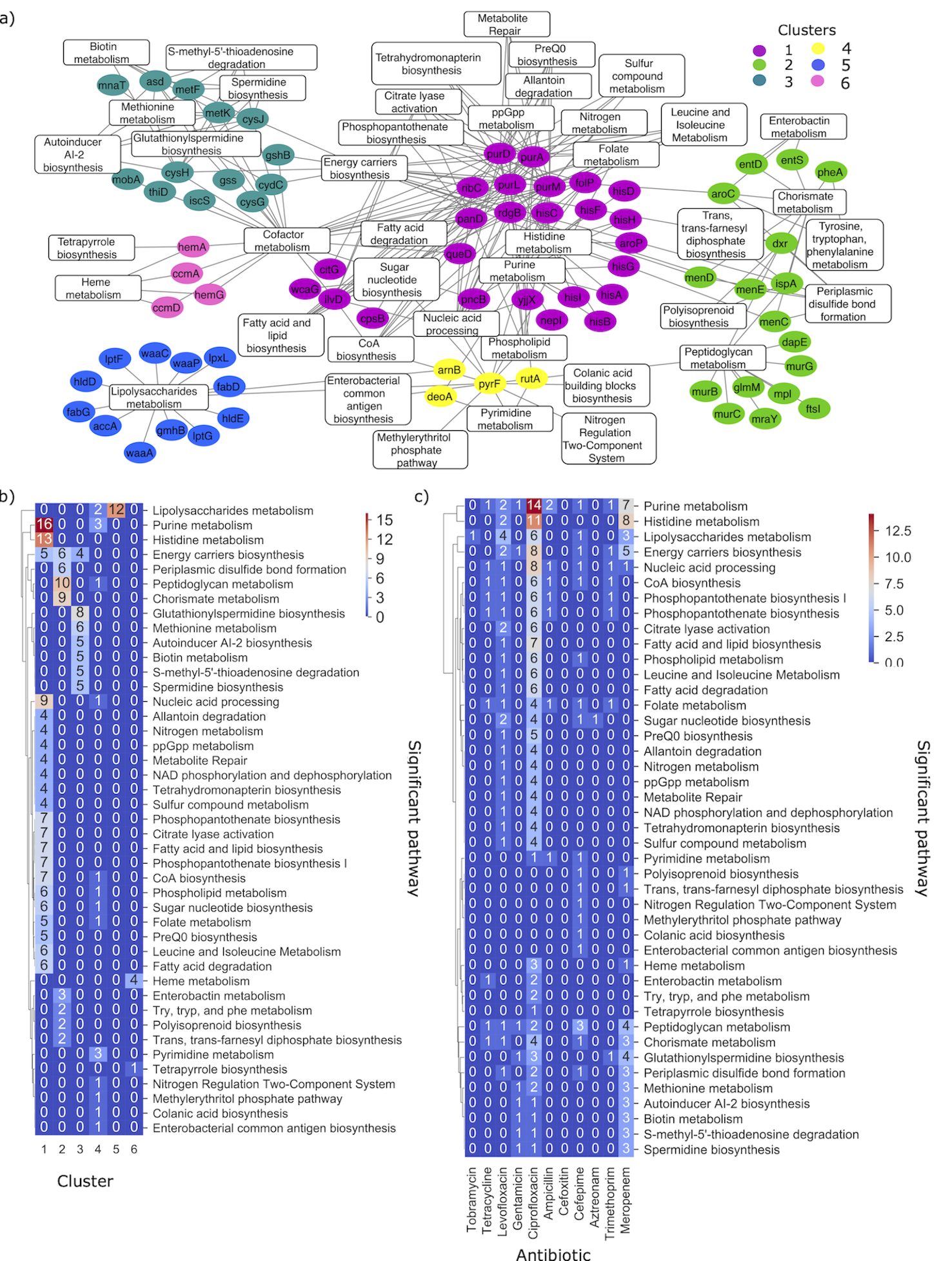

Supplement: FIG S4 [file msystems.00913-20-sf004.tif]

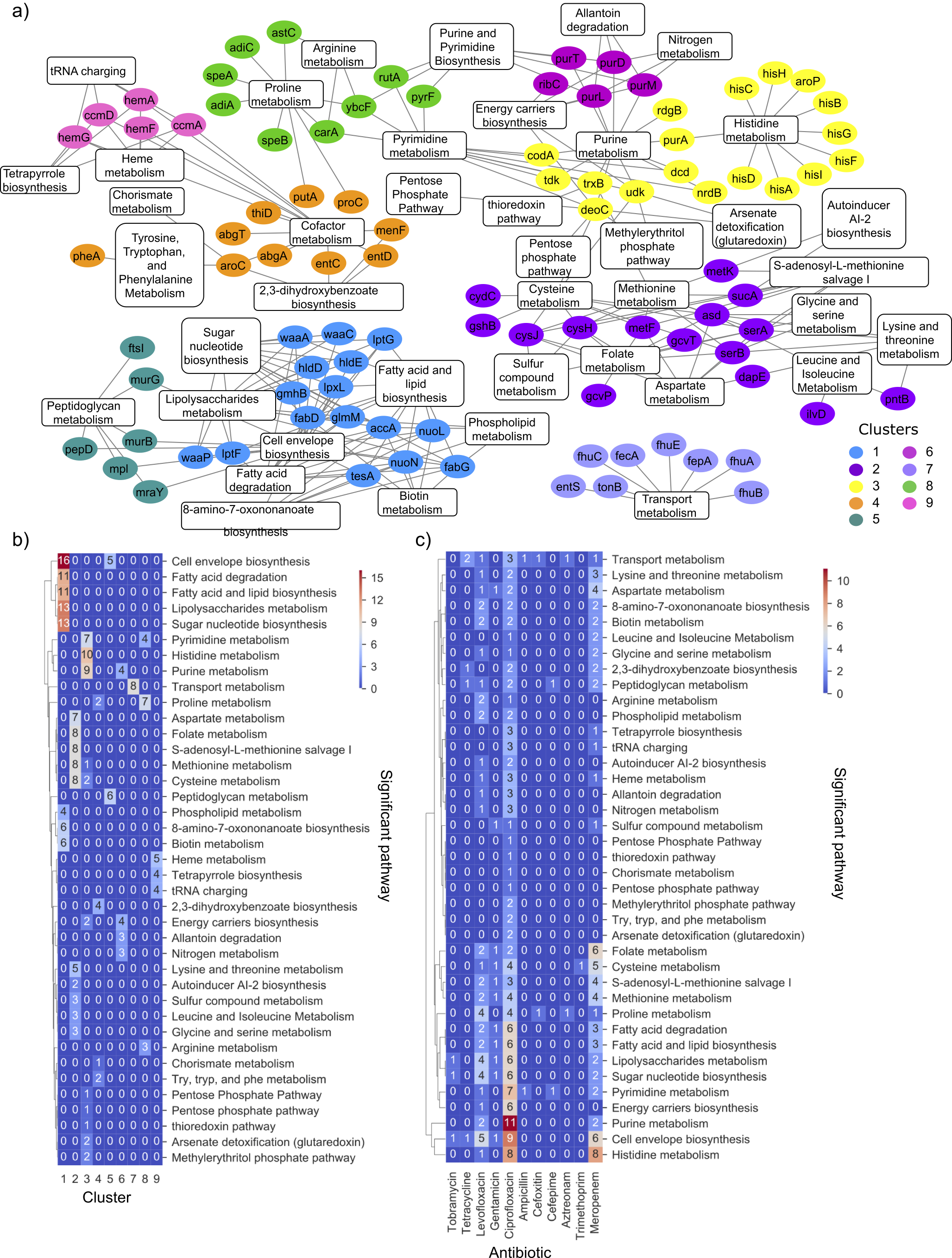

Supplement: FIG S6 [file msystems.00913-20-sf006.tif]

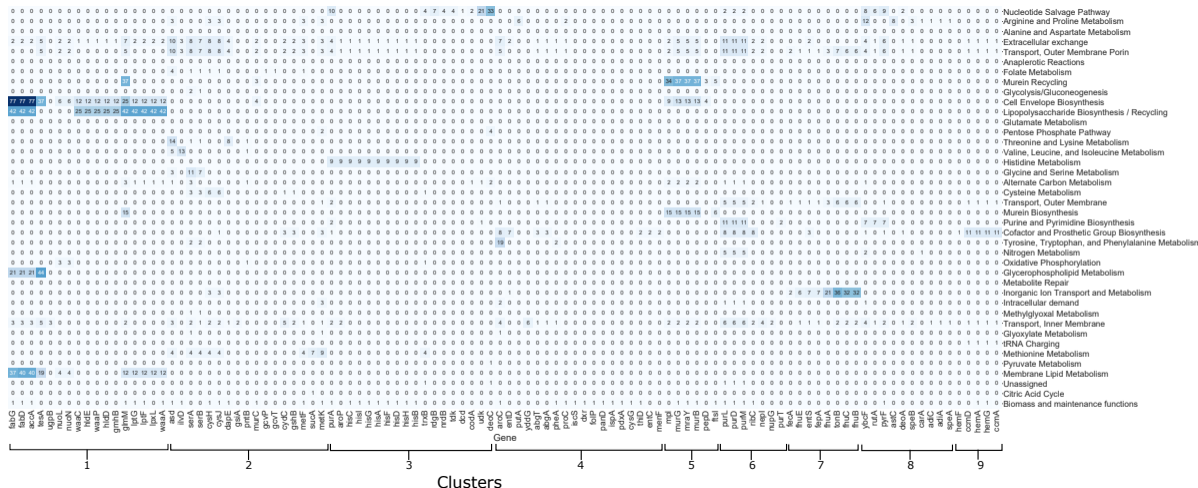

Supplement: FIG S7 [file msystems.00913-20-sf007.pdf]
