# Supplementary material for: Genome-Scale Metabolic Models and Machine Learning Reveal Genetic Determinants of Antibiotic Resistance in Escherichia coli and Unravel the Underlying Metabolic Adaptation Mechanisms
Source: mSystems. 2021 Aug 3;6(4):e00913-20. doi: 10.1128/mSystems.00913-20 (PMC8409726; doi:10.1128/mSystems.00913-20)
Supplement: FIG S2 [file msystems.00913-20-sf002.pdf]

(a)

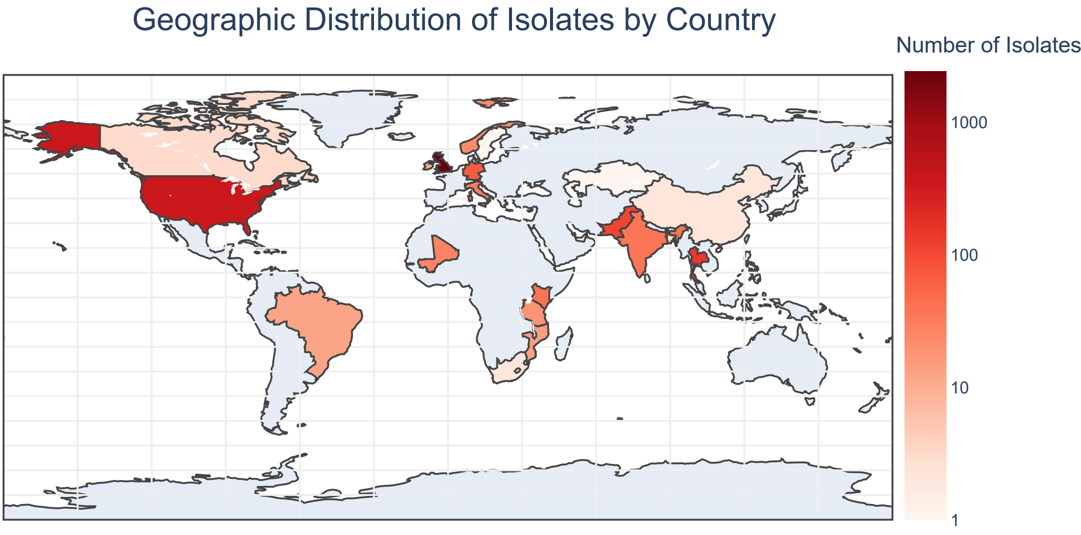

Geographic Distribution of Isolates by Country
Number of Isolates
1000
100
10
1

(b)

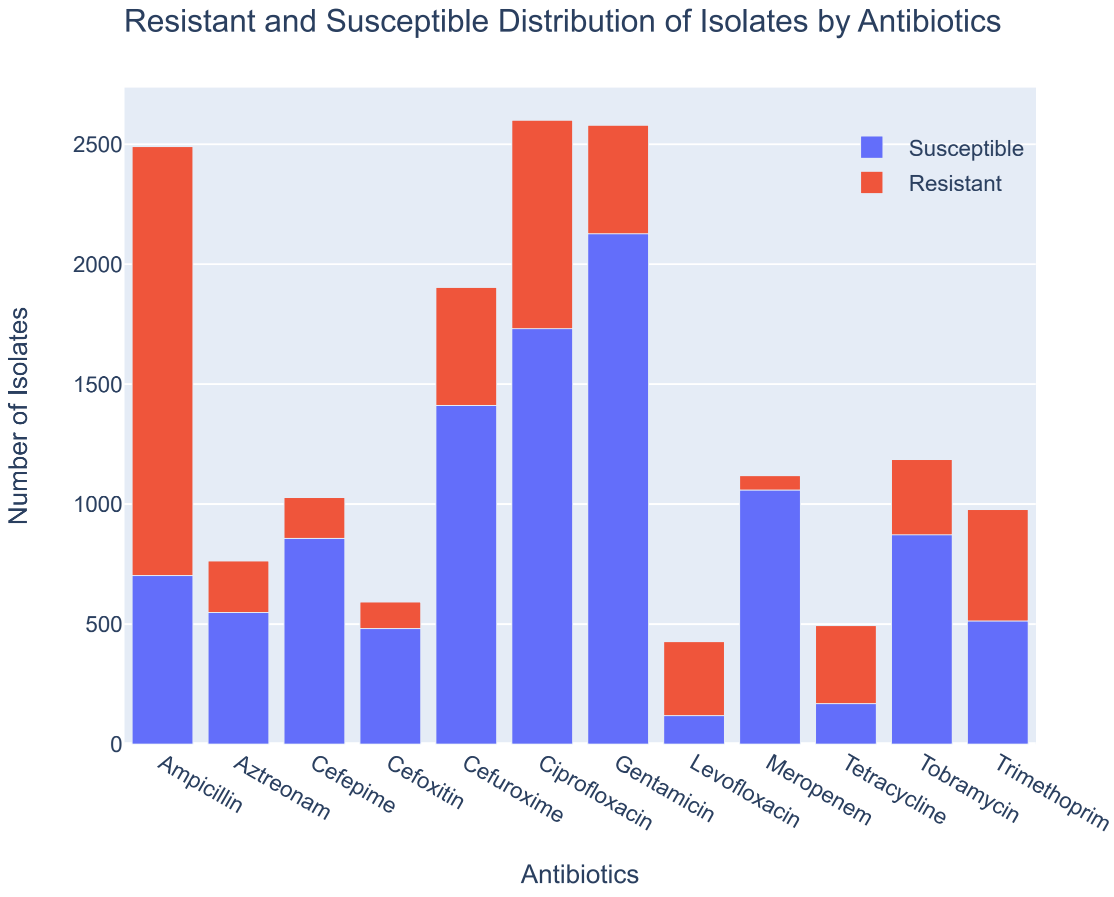

Resistant and Susceptible Distribution of Isolates by Antibiotics
Susceptible
Resistant
Number of Isolates
2500
2000
1500
1000
500
0
Ampicillin
Aztreonam
Cefepime
Cefoxitin
Cefuroxime
Ciprofloxacin
Gentamicin
Levofloxacin
Meropenem
Tetracycline
Tobramycin
Trimethoprim
Antibiotics
