# Supplementary material for: Genome-Scale Metabolic Models and Machine Learning Reveal Genetic Determinants of Antibiotic Resistance in Escherichia coli and Unravel the Underlying Metabolic Adaptation Mechanisms
Source: mSystems. 2021 Aug 3;6(4):e00913-20. doi: 10.1128/mSystems.00913-20 (PMC8409726; doi:10.1128/mSystems.00913-20)
Supplement: FIG S5 [file msystems.00913-20-sf005.pdf]

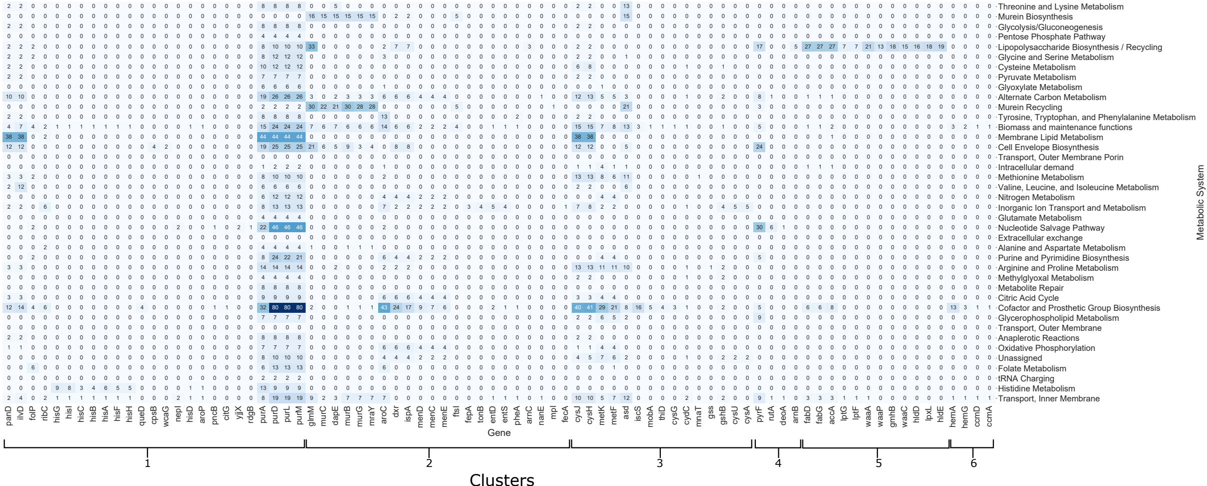
Threonine and Lysine Metabolism
Murein Biosynthesis
Glycolysis/Gluconeogenesis
Pentose Phosphate Pathway
Lipopolysaccharide Biosynthesis / Recycling
Glycine and Serine Metabolism
Cysteine Metabolism
Pyruvate Metabolism
Glyoxylate Metabolism
Alternate Carbon Metabolism
Murein Recycling
Tyrosine, Tryptophan, and Phenylalanine Metabolism
Biomass and maintenance functions
Membrane Lipid Metabolism
Cell Envelope Biosynthesis
Transport, Outer Membrane Porin
Intracellular demand
Methionine Metabolism
Valine, Leucine, and Isoleucine Metabolism
Nitrogen Metabolism
Inorganic Ion Transport and Metabolism
Glutamate Metabolism
Nucleotide Salvage Pathway
Extracellular exchange
Alanine and Aspartate Metabolism
Purine and Pyrimidine Biosynthesis
Arginine and Proline Metabolism
Methylglyoxal Metabolism
Metabolite Repair
Citric Acid Cycle
Cofactor and Prosthetic Group Biosynthesis
Glycerophospholipid Metabolism
Transport, Outer Membrane
Anaplerotic Reactions
Oxidative Phosphorylation
Unassigned
Folate Metabolism
tRNA Charging
Histidine Metabolism
Transport, Inner Membrane
Metabolic System
panD ilvD folP ribC hisG hisI hisC hisB hisA hisF hisH queD cpsB wcaG nepI hisD aroP pncB citG yjjX rdgB purA purD purL purM glmM murC dapE murB murG mraY aroC dxr ispA menD menC menE ftsI fepA tonB entD entS pheA arnC nanE mpl fecA cysJ cysH metK metF asd iscS mobA thiD cysG cydC mnaT gss gshB cysU cysA pyrF rutA deoA arnB fabD fabG accA lptG lptF waaA waaP gmhB waaC hldD lpxL hldE hemA hemG ccmD ccmA
Gene
1
2
3
4
5
6
Clusters
