# Supplementary material for: Genome-Scale Metabolic Models and Machine Learning Reveal Genetic Determinants of Antibiotic Resistance in Escherichia coli and Unravel the Underlying Metabolic Adaptation Mechanisms
Source: mSystems. 2021 Aug 3;6(4):e00913-20. doi: 10.1128/mSystems.00913-20 (PMC8409726; doi:10.1128/mSystems.00913-20)
Supplement: FIG S3 [file msystems.00913-20-sf003.pdf]

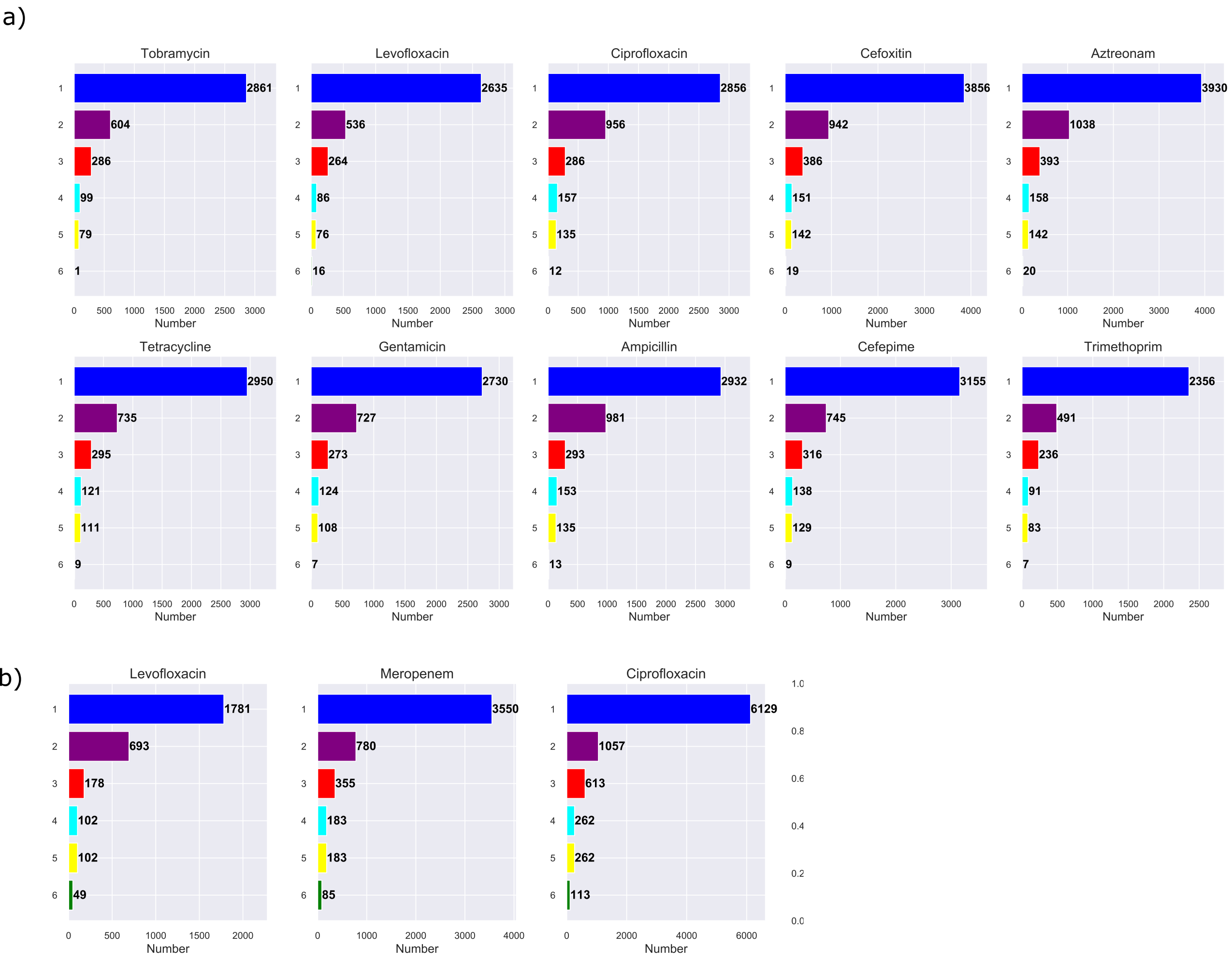

1. Total number of features (k-mer or SNP) in the AMR model
2. Number of unique genes corresponding to the features in the AMR model
3. Number of features in the top 10% of features in the AMR model
4. Number of unique genes corresponding to the top 10% of features in the AMR model
5. Number of genes with functional annotational which correspond to features in the top 10% of features in the AMR model
6. Number of genes in the genome scale model which correspond to features in the top 10% of features in the AMR model
